# (1→3)-α-d-Glucan from the Pink Oyster Mushroom (*Pleurotus djamor*): Structural Features

**DOI:** 10.3390/foods14071272

**Published:** 2025-04-05

**Authors:** Paulina Adamczyk, Iwona Komaniecka, Marek Siwulski, Kamila Wlizło, Adam Junka, Artur Nowak, Dariusz Kowalczyk, Adam Waśko, Jolanta Lisiecka, Michał Grzymajło, Adrian Wiater

**Affiliations:** 1 Department of Industrial and Environmental Microbiology, Institute of Biological Sciences, Faculty of Biology and Biotechnology, Maria Curie-Skłodowska University, Akademicka 19, 20-033 Lublin, Poland; paulina.adamczyk@mail.umcs.pl (P.A.); kamila.wlizlo@mail.umcs.pl (K.W.); artur.nowak@mail.umcs.pl (A.N.); 2 Department of Genetics and Microbiology, Institute of Biological Sciences, Faculty of Biology and Biotechnology, Maria Curie-Skłodowska University, Akademicka 19, 20-033 Lublin, Poland; iwona.komaniecka@mail.umcs.pl; 3 Department of Vegetable Crops, Faculty of Agriculture, Horticulture and Biotechnology, Poznań University of Life Sciences, Dąbrowskiego 159, 60-594 Poznań, Poland; marek.siwulski@up.poznan.pl (M.S.); jolanta.lisiecka@up.poznan.pl (J.L.); 4 “P.U.M.A.”, Platform for Unique Model Application, Department of Pharmacy, Wroclaw Medical University, Borowska 211, 50-534 Wroclaw, Poland; adam.junka@umw.edu.pl; 5 Department of Biochemistry and Food Chemistry, Faculty of Food Sciences and Biotechnology, University of Life Sciences in Lublin, Skromna 8, 20-704 Lublin, Poland; dariusz.kowalczyk@up.lublin.pl; 6 Department of Biotechnology, Microbiology and Human Nutrition, Faculty of Food Science and Biotechnology, University of Life Sciences in Lublin, Skromna 8, 20-704 Lublin, Poland; 7 Department of Polymer Engineering and Technology, Faculty of Chemistry, Wrocław University of Science and Technology (WUST); Wyb. Wyspiańskiego 27, 50-370 Wrocław, Poland; michal.grzymajlo@pwr.edu.pl

**Keywords:** *Pleurotus djamor*, pink oyster mushroom, fruiting bodies, (1→3)-α-d-glucan, structural analyses

## Abstract

(1→3)-α-d-Glucan is an important component of the cell wall of most fungi. The polymer has many applications, including as a therapeutic agent in the prevention or treatment of various diseases, as well as a heavy metal sorbent and a component of new materials used in the plastics industry. The presence of (1→3)-α-d-glucan (water-insoluble, alkali-soluble polysaccharide) in the cell wall of *Pleurotus djamor* (pink oyster mushroom) was confirmed using specific fluorophore-labeled antibodies. Therefore, the water-insoluble fraction (WI-ASF) of *P. djamor* B123 fruiting bodies was isolated by alkaline extraction and used for further analyses. The structural features of the WI-ASF were determined by composition analysis, linkage analysis, Fourier transform infrared and Raman spectroscopy, ^1^H and ^13^C nuclear magnetic resonance spectroscopy, scanning electron microscopy, as well as viscosity, specific rotation, and gel permeation chromatography. These studies revealed the presence of glucose units linked by α-glycosidic bonds and scanty amounts of mannose and xylose. Furthermore, methylation analysis of WI-ASF demonstrated that the (1→3)-linked glucopyranose (Glc*p*) is the primary moiety (86.4%) of the polymer, while the 3,4- and 3,6-substituted hexoses are the branching residues of the glucan. The results of chemical and spectroscopic investigations indicated that the analyzed WI-ASF is a (1→3)-linked α-d-glucan type with a molecular weight of 552 kDa.

## 1. Introduction

(1→3)-α-d-Glucans are an essential component of the cell wall of most fungi (exceptions including the yeasts *Saccharomyces cerevisiae* and *Candida albicans*), where they have many biological functions, e.g., structural, nutritive, aggregation-promoting, or virulence factors for some pathogens (e.g., α-glucan from *Aspergillus fumigatus*) [1,2,3]. In comparison to other polymers present in the fungal cell wall, the (1→3)-α-d-glucans are relatively poorly understood and researched. The insoluble nature, the unique crystalline structure, and the presence of α-(1→3)-glycosidic linkages have defined the applicability of this polymer. To date, it has been shown, among other things, that (1→3)-α-d-glucans: (i) constitute a safe, inexpensive and readily available mutanase inducer, enzymes used in dental plaque degradation and prevention of tooth decay in humans and animals; (ii) after chemical modification (e.g., carboxymethylation, sulfation, aminopropylation, or hydroxyethylation) as water-soluble derivatives, they had significant anticancer, immunomodulatory, and antioxidant potential; (iii) can be used in combination with a catalyst (such as lipase) in the esterification reaction of higher fatty acids with alcohols with hydrophobic alkyl chains; (iv) can be an efficient sorbents for the removal of toxic metal ions from aquatic environments; (v) can be used to bind mycotoxins present in animal feed; (vi) are a source of oligosaccharides with prebiotic properties; and (vii) due to their exceptional thermoplastic and texture-forming properties, they are used in the plastics and textile industries [2,4,5,6,7,8,9].

The fruiting bodies of cultivated mushrooms, especially *Pleurotus* spp., are excellent sources of (1→3)-α-d-glucans. As shown in our previous studies, the biomass of these fungi was a source of glucans with anticancer properties [10,11]. An additional benefit is that waste fungal biomass, which is a crucial problem in the cultivation of oyster mushrooms, can be used for this purpose. This approach is in line with the zero-waste concept.

*Pleurotus djamor* (Rumph. ex Fr.) Boedijn is a species from the oyster mushroom group native to South-East Asia and Central America [12]. Salomones et al. [13] reported that the species also occurs in Africa and Australia. In tropical and subtropical regions, it occurs naturally on various species of trees, such as palms, bamboo, rubber trees, and mangoes, and is therefore readily available on the market [12,14,15]. Its cultivation is becoming increasingly popular worldwide due to the pink color of its fruiting bodies (pink oyster mushroom) and its high nutritional and medicinal value. Several studies have confirmed the antioxidant activity of *P. djamor* [16,17,18,19] as well as its broad spectrum of other medicinal activity, including hypoglycemic, anti-cancer, and anti-inflammatory effects [20,21,22]. Antimicrobial activity has also been demonstrated against different taxons of bacteria, e.g., *Escherichia coli*, *Staphylococcus aureus*, *Vibrio cholera*, *Xanthomonas* spp., and *Pseudomonas* spp. [23,24,25]. A positive effect of *P. djamor* on the human gut microbiota was also confirmed [26].

*P. djamor* is quite easy to cultivate due to its fast-growing mycelium and the possibility of using a wide range of waste materials derived from agriculture, forestry, and the food industry [27,28,29,30,31,32]. The fruiting bodies of this species are distinguished by their high dry matter content and very attractive aroma [33,34]. The high protein and the macro and micronutrient content of *P. djamor* accounts for its nutritional value [27,35]. The content of nutritionally important minerals in the fruiting bodies can easily be increased by supplementing the growing medium with some microelements, such as magnesium, zinc, and selenium salts [36,37,38].

As revealed by an analysis of the available articles, the amount of research work on *Pleurotus* (1→3)-α-d-glucans is limited. In this paper, we focus on the detection, isolation, structure, and some physicochemical properties of a water-insoluble (1→3)-α-d-glucan obtained from the pink oyster mushroom *P. djamor* via alkaline extraction.

## 2. Materials and Methods

### 2.1. Fungal Material and Fruiting Conditions

Strain B123 of *Pleurotus djamor* originated from the Mushroom Collection of the Department of Vegetable Crops at the University of Life Sciences in Poznań, Poland.

**Figure 1:**
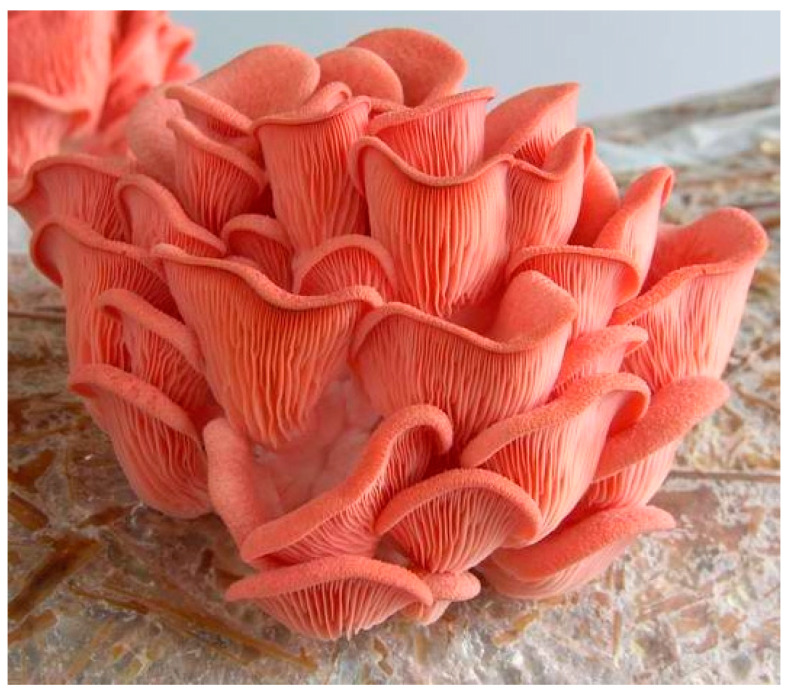
Fruiting bodies of *P. djamor* B123 on an artificial substrate.

Cultivation of *P. djamor* fruiting bodies was carried out in the vegetation hall of the Department of Vegetable Crops at the University of Life Sciences in Poznań. The substrate for cultivation was prepared from a mixture of beech and alder sawdust (1:1, *v*/*v*) with several additives. One kilogram of the substrate contained 300 g of sawdust, 78 g of wheat bran, 20 g of maize flour, 2 g of gypsum, and 600 mL of tap water. The cultivation was carried out in polypropylene bottles (850 mL volume). Each bottle was filled with 500 g of the substrate. The bottles were sterilized at a temperature of 121 °C for 1 h. Mycelium of *P. djamor* on wheat grain was used for substrate inoculation in the amount of 10 g per bottle. Incubation was conducted at 25 °C and 80–85% relative air humidity until the substrate became completely covered with mycelium. Next, the bottles were placed in the cultivation chamber. For fructification, air relative humidity and temperature were maintained at 85–90% and 22 ± 1 °C, respectively. The cultivation was additionally lighted with fluorescent light of 500 lx intensity with a photoperiod regime of 12 h dark and 12 h light. The cultivation chamber was aerated in such a way as to maintain the CO_2_ concentration below 1000 ppm. Fruiting bodies of *P. djamor* were harvested successively as they matured (Figure 1). The yield included whole fruiting bodies. The obtained fruiting bodies were dried in an electric drier at 40 °C to a constant weight.

### 2.2. WI-ASF Isolation

The water-insoluble, alkali-soluble fraction (WI-ASF) was isolated from the fruiting bodies of *P. djamor* B123 according to the method described previously [39]. Briefly, the dried fruiting bodies (100 g) were milled into powder using a high-speed blade mill WŻ-1 (Research Institute for the Bakery Industry, Bydgoszcz, Poland) and extracted successively with 2 L of methanol, NaCl (0.9%), hot water, Na_2_CO_3_ (5%), and finally, with 1 M NaOH containing 0.02% sodium borohydride (NaBH_4_, a reducing agent) for 24 h at room temperature (all reagents were form POCh, Gliwice, Poland). The final extract was neutralized with 1 M HCl under constant mixing, and the precipitated fraction (WI-ASF) was repeatedly washed with water, collected by centrifugation, and lyophilized. The yield of WI-ASF isolated from fruiting bodies of *P. djamor* B123 was 2.8 g/100 g of dry mass.

### 2.3. Sugar Composition and Methylation Analysis of WI-ASF from P. djamor

The WI-ASF (2 mg) was acid hydrolyzed using 2 M trifluoroacetic acid (TFA, Sigma-Aldrich, Saint Luis, MO, USA) at 100 °C for 4 h. Liberated monosaccharides were reduced by using sodium borodeuteride (NaBD_4_, Sigma-Aldrich, Saint Luis, MO, USA), and obtained products were acetylated with pyridine-acetic anhydride mixture (Sigma-Aldrich, Saint Luis, MO, USA) (1:1, *v:v*) at 85 °C for 30 min to obtain alditol acetates [40]. For quantitative analyses, inositol was used as an internal standard.

Linkage analysis of sugars in the polysaccharide was performed with the method described by Hakomori [41]. Briefly, the sample (5 mg) was dissolved in 0.5 mL dry dimethylsulfoxide (DMSO, Sigma-Aldrich, Saint Luis, MO, USA), then 0.5 mL of dimsyl Na (de-protonating agent, made “on-side” from DMSO and NaH) was added, and methylation occurred in a presence of methyl iodide (1 mL, Sigma-Aldrich, Saint Luis, MO, USA). An excess of reagents was removed in a stream of nitrogen, and the reaction was stopped by adding water. The partly methylated polymer obtained was extracted into chloroform, dried in a stream of nitrogen, and acid hydrolyzed (2 M TFA, 100 °C for 4 h). The partly methylated sugar monomers were reduced and acetylated as above.

The absolute configuration of the monosaccharides was established by analysis of acetylated R-(−)2-butylglycosides according to the procedure described by Gerwig and co-workers [42].

All sugar derivatives were analyzed by GC-MS using a 7890A gas chromatograph connected to an MSD 5975C (inert XL EI/CI MSD) detector (Agilent Technologies, Santa Clara, CA, USA) equipped with an HP-5MS column (30 m × 0.25 mm, film thickness 0.25 µm; Agilent J&W GC Columns, Agilent, Santa Clara, CA, USA). Helium was a carrier gas with a flow rate of 1 mL/min. The temperature program was as follows: from 150 °C (3 min) raised to 320 °C at 5 °C min^−1^, and the final temperature was maintained for 10 min. Sugar derivatives were identified based on their retention times and characteristic mass spectra, using Enhanced Data Analysis software, version 5.02.09 (Agilent Technologies, Santa Clara, CA, USA).

### 2.4. Miscellaneous Methods

Immunofluorescent labeling of the (1→3)-α-d-glucan in the cell wall of *P. djamor* B123 was carried out using specific antibodies, i.e., mouse IgM MOPC-104E and goat anti-mouse IgM labeled with a fluorescent dye [43]. Briefly, fresh mycelium of *P. djamor* B123 on Lab-Tek II Chamber slides (Nunc, Rochester, NY, USA) was fixed with a 3% (*v*/*v*) formaldehyde solution in distilled water at 65 °C for 30 min. The fixed fungal cells were washed three times in PBS (Phosphate Buffered Saline, pH 7.4) before being infiltrated with 1% (*v*/*v*) Tween 20 in PBS (PBS-T). To detect the presence of (1→3)-α-d-glucan, a 150 µL solution of mouse IgM MOPC-104E (0.1 mg/mL in PBS) (Sigma, Saint Louis, MO, USA) as a primary antibody and 150 µL Alexa Fluor 488 goat anti-mouse IgM (µ-chain specific) (0.1 mg/mL in PBS) (Sigma, Saint Louis, MO, USA) as a secondary antibody were used. Fungal material was incubated with the primary antibodies overnight at 4 °C in a wet chamber. Incubation with the secondary antibodies was performed for 2 h in the dark at 37 °C. Before observation the antibody-labeled cells were rinsed three times with PBS. The (1→3)-d-α-glucan was observed using an Olympus BX 51 microscope (Olympus, Tokyo, Japan) (excitation at 470/500 nm and emission at 525/550 nm).

The polysaccharide average molecular weight (Mw) was determined by gel permeation chromatography (GPC) using a Sepharose CL-6B column (Sigma-Aldrich, Saint Luis, MO, USA) (0.7 cm × 90 cm), as previously described by Choma et al. [44]. The (1→3)-α-d-glucan (2 mg) was dissolved in 0.5 mL of 1 M sodium hydroxide solution (POCh, Gliwice, Poland). The column was eluted with 1 M NaOH at a flow rate of 0.3 mL/min. The separation was completed at room temperature. Due to the strongly alkaline character of eluate, the total content of carbohydrates was determined using the phenol-sulfuric acid assay according to the Dubois method [45]. The column was calibrated using dextrans of known molecular masses.

The Fourier transform infrared (FTIR) spectrum between 400 and 4000 cm^−1^ was recorded using a PerkinElmer FTIR spectrophotometer Model 1725X (PerkinElmer Corp., Norwalk, CT, USA). A specimen was prepared using the KBr-disk method.

The Fourier transform Raman (FT-Raman) spectrum was recorded using a Nicolet NXR FT-Raman module for a Nicolet 6700 Fourier transform infrared spectroscopy bench that used a InGaAs detector and CaF_2_ beam splitter (Thermo Scientific, Madison, WI, USA). The sample in the form of powder was placed in stainless cubes and was illuminated using an Nd:YAG excitation laser operating at 1064 nm. The maximum laser power was 0.6 W. The spectrum was recorded in the range of 3700–150 cm^−1^, and each spectrum averaged 200 scans with 8 cm^−1^ resolution. The analyzed spectra were averaged over three registered spectra.

The specific rotation ${\left[\alpha \right]}_{D}^{25}$ (*c* 1 M sodium hydroxide) was measured at 589 nm in a Perkin-Elmer Automatic Polarimeter model 341 LC (Wellesley, MA, USA). The viscosity of polysaccharides (*c* 1 M sodium hydroxide) was measured using a Brookfield model DV 3 viscometer (Stoughton, MA, USA) at 20 °C.

One-dimensional ^1^H and ^13^C NMR spectra were recorded with a Bruker Avance (300 MHz, Billerica, MA, USA) spectrometer at 60 °C (333 K) using standard Bruker software (TopSpin 3.2). The WI-ASF sample (5 mg) was dissolved in 0.7 mL of 1 M NaOD in D_2_O (Sigma-Aldrich, Saint Louis, MO, USA). The ^1^H and ^13^C resonances were shifted to internal acetone (*δ*_H_ 2.225 ppm/*δ*_C_ 31.07 ppm).

Scanning electron microscopy (SEM) was conducted using an EVO Zeiss MA microscope. To prepare the (1→3)-α-d-glucan powder sample for SEM, a small amount of glucan lyophilizate was applied to a conductive double-sided adhesive tape affixed to a standard SEM stub. Excess unbound material was gently removed using a stream of compressed air. To ensure electrical conductivity and minimize charging effects, the sample was coated with a thin layer of an Au:Pd mixture using a Vacuum Sputter Coater. Observations were performed at an accelerating voltage of 10 kV with a working distance of 10 mm. Images were acquired at magnifications of 100×, 600×, and 2020× facilitating detailed assessment of the morphology and surface structure of the (1→3)-α-d-glucan samples. All measurements were carried out in high-vacuum conditions to ensure optimal image quality.

## 3. Results and Discussion

**Figure 2:**
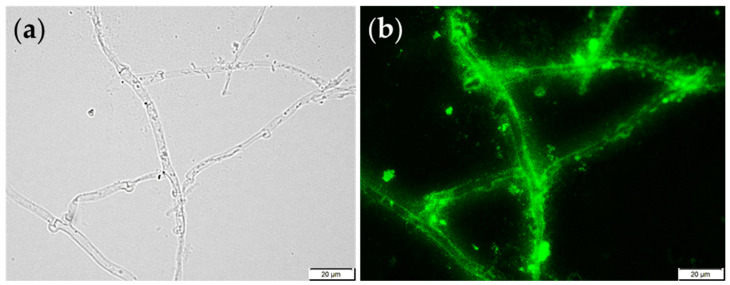
Immunofluorescent labeling of (1→3)-α-d-glucan in the mycelium of *P. djamor* B123 using specific labeled antibodies. (**a**) View in the light microscopy; (**b**) The same filaments in fluorescence microscopy. At least 10 preparations were observed, and representative images were chosen for presentation. Scale bar corresponds to 20 μm.

The presence of (1→3)-α-d-glucan in the cell wall of *P. djamor* B123 was confirmed using specific antibodies, i.e., primary mouse IgM MOPC-104E and goat anti-mouse IgM labeled with fluorescent dye AlexaFluor 488. Figure 2 shows the results of this investigation. The prominent green luminescence seen in panel **b** indicates the existence of (1→3)-α-d-glucan in the cell wall structure of the *P. djamor* filaments. As can be seen, the (1→3)-α-d-glucan is found along the entire length of the filament, suggesting that this polymer plays an important role in the organization of the fungal cell wall. Numerous studies indicate that (1→3)-α-d-glucan is differentially localized in the fungal cell wall. Studies carried out by Grün in 2003 [46] showed that this polymer is located under the layer of β-glucan, just above the cytoplasmic membrane. However, as suggested by recent reports on the fungal cell wall structure, the position of (1→3)-α-d-glucans is quite variable and species-dependent [1,47]. They can be found in the outer layers of the cell wall (in *Histoplasma capsulatum*) or as part of a complex polysaccharide network under the protein-polysaccharide layer (in *Aspergillus fumigatus*). Furthermore, the distribution of (1→3)-α-d-glucans within the cell wall is influenced by the developmental form of the fungus, i.e., spores or vegetative mycelium, and the type of microorganism culture [47,48].

The WI-ASF used for further analysis of structural characteristics was isolated via alkaline extraction from the fruiting bodies of *P. djamor* B123 grown on artificial medium (Figure 1). The alkali-soluble polysaccharide material (WI-ASF) was obtained with a yield of 2.8% of dry mass. The WI-ASF contained mainly carbohydrates (97.5% of isolated material), as estimated by Dubois method [45], and only traces of protein (0.33%) were detected, as determined using the Bradford method [49].

**Figure 3:**
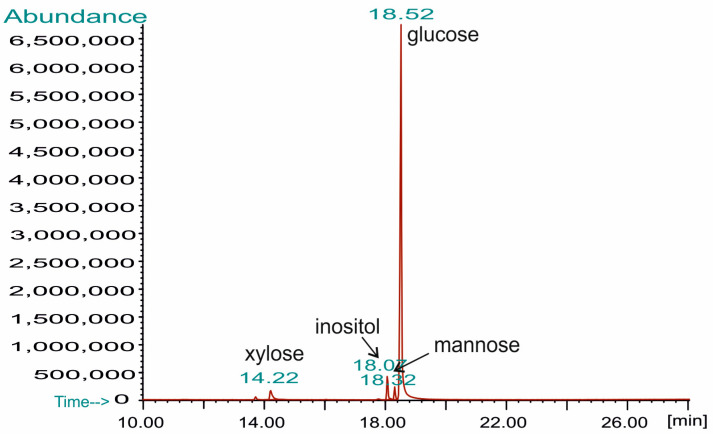
GC-MS chromatogram of monosaccharides released from *P. djamor* WI-ASF. Inositol was used as an internal standard.

The GC-MS analysis of monosaccharides liberated by the acid hydrolysis of the WI-ASF isolated from the fruiting bodies of *P. djamor* showed that the isolated material was composed predominantly of glucose (93.5%), suggesting that this is a glucan polymer. Only small amounts of the other sugars were detected, namely xylose (4.0%) and mannose (2.5%) (Figure 3). All the monosaccharides released have been shown to have the d absolute configuration [42].

The methylation analysis of the WI-ASF showed that (1→3)-linked Hex*p* (identified as (1→3)-linked glucose) is the major chain constituent (86.4%), while (1→4)-linked Hex*p* (4.5%) is the minor one (Table 1). It can be assumed that the isolated polysaccharide is (1→3)-glucan, with a rather short side chains composed of 4-linked hexose. The analyzed WI-ASF also contained two types of doubly substituted glucose residues, i.e., 3,4)-Hex*p* and 3,6)-Hex*p*, representing the main branching points of the (1→3)-linked polysaccharide chain. Moreover, fully methylated (terminal) xylose (2.7%) and hexose (2.1%) as well as (1→2)-linked pentose derivatives were also present.

In summary, the composition analyses indicated that the monosaccharide residues (almost exclusively of α-d-glucose) in the WI-ASF isolated from the fruiting bodies of *P. djamor* are mainly (1→3)-linked.

**Figure 4:**
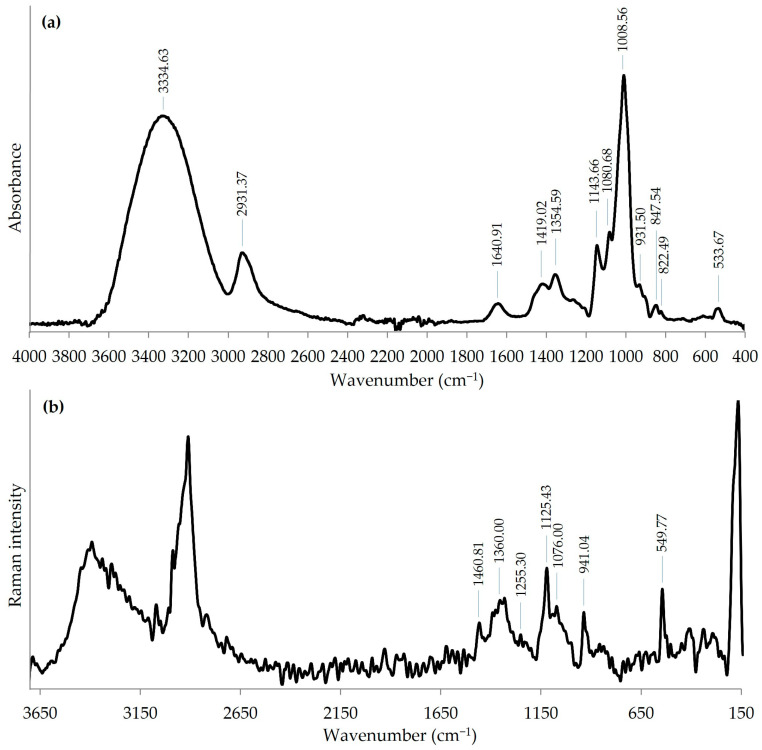
FTIR (**a**) and FT-Raman (**b**) spectra of WI-ASF extracted from the fruiting bodies of *P. djamor*.

The FTIR spectrum of the WI-ASF extracted from the *P. djamor* fruiting bodies is shown in Figure 4a. The wavelength of the FTIR spectrum was set in the range of 400–4000 cm^−1^. The strong and broad band at 3334 cm^−1^ can be attributed to O–H stretching vibrations, and the weaker absorption band at 2931 cm^−1^ can be assigned to C–H vibrations. Furthermore, the peaks in the region of 950–1200 cm^−1^ attributed to the stretching vibrations of the pyranose ring and C-O-C and C-O-H moieties are characteristic for polysaccharides [50,51]. The WI-ASF showed absorption peaks at 931.50, 847.54, and 822.49 cm^−1^. These peaks are characteristic of (1→3)-α-d-glucans [52]. In particular, the symmetrical band at 822.49 cm^−1^ is exclusively associated with (1→3)-α-linkages [53]. The low intense band visible at about 1640 cm^−1^ (derived from Amide I) may be indicative for the presence of very small amounts of proteins in the sample. The (1→3)-α-structure of the WI-ASF obtained from *P. djamor* was also confirmed by Raman spectroscopy (Figure 4b). Bands characteristic of polysaccharides are seen at 1460.81, 1360.00, 1255.30, 1125.43, and 1076.00 cm^−1^. In turn, the intense and sharp bands at 941.04 and 549.77 cm^−1^ confirmed (1→3)-α-d-glucan as the major component of the WI-ASF [54,55].

**Figure 5:**
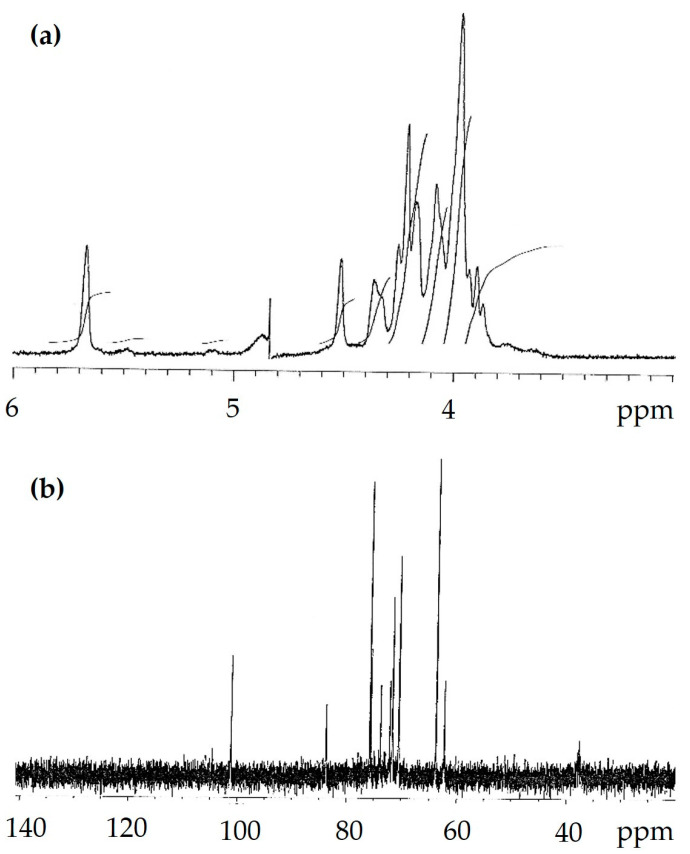
^1^H (**a**) and ^13^C NMR (**b**) spectra of WI-ASF obtained from the fruiting bodies of *P. djamor*.

To confirm the results of the compositional and spectral analyses of the WI-ASF and establish the anomeric configuration of the *P. djamor* polysaccharide, ^1^H and ^13^C NMR spectra were recorded in 1 M NaOD in D_2_O. The 1D NMR spectra are shown in Figure 5. The only spin system identified was characteristic of α-d-glucose. The low-field anomeric proton signal (H-1) at δ 5.62 ppm and the anomeric carbon signal (C-1) at δ 101.2 ppm are typical of the α-anomer of (1→3)-linked glucose [56]. Furthermore, the intense and sharp peak near 71.7 ppm and smaller ones at 83.8 and 62.4 ppm correspond, respectively, to C-2, C-3 and C-6 carbons of (1→3)-α-d-glucan [55].

**Figure 6:**
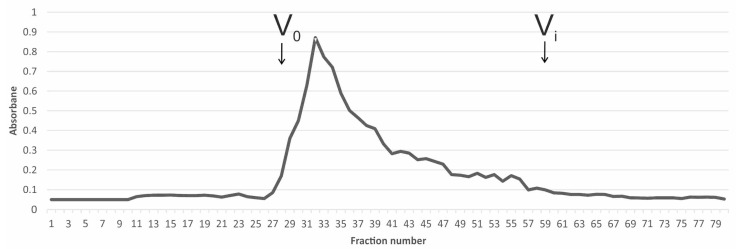
Elution profile (GPC) of the *P. djamor* WI-ASF at Sepharose CL-6B washed with 1.0 M NaOH. V_0_—dead volume; V_i_—inside volume.

Gel permeation chromatography (GPC) allowed the average molecular weight (Mw) of the (1→3)-α-d-glucan to be determined (Figure 6). The polysaccharide preparation dissolved in 1.0 M NaOH and fractionated on a Sepharose CL-6B column exhibited a single broad peak, giving an average Mw of about 552 kDa in the maximum point. This result indicates that polysaccharide chains are long, and more than 3400 sugar subunits were counted. (1→3)-α-d-glucan with similar Mw (560 kDa) was also isolated from the fruiting bodies of *Agrocybe cylindracea* [57]. Molecular weight estimation of the alkali-soluble polysaccharide from *Boletus edulis* showed two peaks, a main at about 850 kDa and a smaller broad peak with a maximum at about 92 kDa [57]. High and low molecular weight fractions, i.e., 2900 and 110.3 kDa, and 2300 and 20.3 kDa, were also detected for alkaline extracts obtained from *Pleurotus ostreatus* and *P. eryngii* fruiting bodies, respectively [55].

The specific optical rotation (${\left[\alpha \right]}_{D}^{25}$) of the WI-ASF was +220° (*c* 1.0, 1 M NaOH). The high and positive value of the optical rotation was comparable to (1→3)-α-d-glucans from *Aspergillus* spp. (from +216° to +384°), *Cerrena unicolor* (+206°), *Agrocybe cylindracea* (+195°), and *Lentinus edodes* (+193.5°) [2,53,58]. The WI-ASF viscosity value oscillated around 11.45 mPa·s, and was similar to values (from 12.2 to 17.0 mPa·s) measured for (1→3)-α-d-glucans isolated from *Aspergillus* spp. biomass, including the linear pseudonigeran (8.8 mPa·s) from *A. niger* [2].

Taking all the above data together, it can be concluded that the WI-ASF extracted from the fruiting bodies of *P. djamor* contains mainly (1→3)-α-d-glucan.

**Figure 7:**
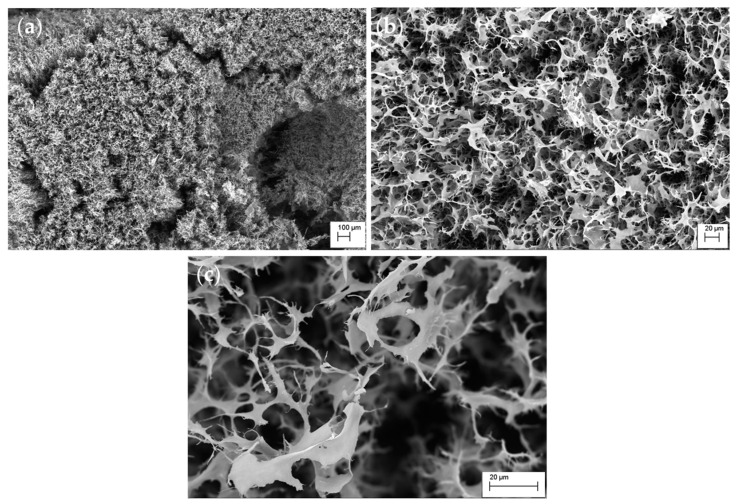
SEM of (1→3)-α-d-glucan from the fruiting bodies of *P. djamor*: (**a**) ×100, (**b**) ×600, (**c**) ×2020 magnification.

The scanning electron microscopy (SEM) analysis was used to complete the (1→3)-α-d-glucan characterization. The SEM images show the polysaccharide microstructure at magnifications of 100×, 600×, and 2020× (Figure 7). At 100× magnification, the image reveals the overall spatial arrangement of (1→3)-α-d-glucan, organized as a heterogeneous fibrous network (Figure 7a). This network results from intertwined polysaccharide fibers that constitute (1→3)-α-d-glucan. Increasing the magnification to 600× facilitates detailed observation of the individual fibers forming the (1→3)-α-d-glucan structure (Figure 7b). The image shows interconnected irregular fibers creating a three-dimensional matrix with uneven pores. Notably, the fibers exhibit varying diameters, with some appearing thicker and others more thread-like. At the highest magnification of 2020×, the SEM image highlights micropores and the surface intricacies of the fibers (Figure 7c). The (1→3)-α-d-glucan exhibit porous architecture, which may influence its functional properties, such as water retention or interaction with other substances. The surface displays numerous indentations and pores, indicating a substantial specific surface area. In summary, these SEM images illustrate that the (1→3)-α-d-glucan extracted from *P. djamor* forms a complex fibrous network with irregular porosity.

## 4. Conclusions

This study showed that (1→3)-α-d-glucan is the structural component of the cell wall of pink oyster mushroom *P. djamor*. All the analyses performed confirm that the water-insoluble, alkali-soluble polysaccharidic material extracted from the fruiting bodies of *P. djamor* B123 contained mainly (1→3)-α-d-glucan. A number of reports indicate that (1→3)-α-d-glucan can be used in a wide range of applications [2,59,60,61]; therefore, new cheap and safe sources of this polymer are being sought. The occurrence of (1→3)-α-d-glucan in *P. djamor* biomass opens up the possibility of extracting this polymer on a larger scale from cultivated oyster mushrooms, especially those considered a waste product.

## Figures and Tables

**Table 1:** Results of the methylation analysis of the *P. djamor* WI-ASF polysaccharide.

Methylated Sugar	Linkage Type	Mol (%)
2,3,4-*O*-Me_3_-pentose	*t*Pen*p*-(1→	2.7
3,4-*O*-Me_2_-pentose	→2)-Pen*p*-(1→	1.3
2,3,4,6-*O*-Me_4_-hexose	*t*Hex*p*-(1→	2.1
2,4,6-*O*-Me_3_-hexose	→3)-Hex*p*-(1→	86.4
2,3,6-*O*-Me_3_-hexose	→4)-Hex*p*-(1→	4.5
2,6-*O*-Me_2_- hexose	→3,4)-Hex*p*-(1→	1.2
2,4-*O*-Me_2_- hexose	→3,6)-Hex*p*-(1→	1.8

The polysaccharide preparation was methylated before being hydrolyzed, reduced, and acetylated. Therefore, the methylated sugars mentioned refer to permethylated alditol acetates identified by GC–MS (e.g., 2,3,4,6-*O*-Me_4_-hexose refers to 1,5-di-*O*-acetyl-2,3,4,6-tetra-*O*-methyl-hexitol). *t*–terminal residue.

## Data Availability

The original contributions presented in this study are included in the article. Further inquiries can be directed to the corresponding authors.

## Abbreviations

The following abbreviations are used in this manuscript:

WI-ASF	Water-insoluble alkali-soluble fraction
PBS	Phosphate buffered saline
SEM	Scanning electron microscopy
TFA	Trifluoroacetic acid
GPC	Gel permeation chromatography
FT-IR	Fourier transform infrared spectroscopy
FT-Raman	Fourier transform Raman
